# Changes in the Soil Fungal Community Mediated by a *Peganum harmala* Allelochemical

**DOI:** 10.3389/fmicb.2022.911836

**Published:** 2022-06-16

**Authors:** Kai Shi, Hua Shao

**Affiliations:** ^1^State Key Laboratory of Desert and Oasis Ecology, Xinjiang Institute of Ecology and Geography, Chinese Academy of Sciences, Ürümqi, China; ^2^University of the Chinese Academy of Sciences, Beijing, China; ^3^Research Center for Ecology and Environment of Central Asia, Xinjiang Institute of Ecology and Geography, Chinese Academy of Sciences, Ürümqi, China

**Keywords:** allelopathy, harmaline, *Peganum harmala*, plant-microbe interactions, soil fungi

## Abstract

Plants can release phytotoxic allelochemicals into the environment, not only to suppress other plants’ growth, but also alter community structure of soil microbiota, however, the mechanism are often complicated. We designed a consecutive cultivation procedure to evaluate the allelopathic effect of harmaline, the major active allelochemical produced by the desert plant *Peganum harmala*, on soil microorganisms. Harmaline was added to the soil at 20 μg/g, and after five generations of cultivation, the Chao1, Pielou, Shannon and Simpon indexes changed significantly. In particular, the relative abundances of the dominant fungi, *Alternaria* sp. and *Fusarium* sp., declined drastically by 84.90 and 91.90%, respectively. Further *in vitro* bioassays confirmed that harmaline indeed suppressed growth of 6 *Alternaria* and *Fusarium* strains isolated from *P. harmala* rhizosphere soil. We thus suspect that *P. harmala* might produce harmaline as an effective carry-on pesticide to defend against general pathogens such as *Alternaria* sp. and *Fusarium* sp. and favor itself. Our consecutive cultivation procedure has successfully magnified the core signals from the chaotic data, implying that it can be applied to measure the effects of other allelochemicals on soil microbiota.

## Introduction

Biological invasion has caused great damage worldwide. In the past decades, a number of hypotheses have attempted to elucidate the mechanism of biological invasion, and allelopathy is considered to contribute to the establishment of dominance of certain invasive species (Callaway and Ridenour, 2004; Shao et al., 2013). These species either directly release allelochemicals into the air or soil matrix or indirectly alter the physiochemical properties of the surrounding microenvironments as well as soil microorganisms to negatively affect the growth and reproduction of native species. A recent meta-analysis focusing on direct allelopathic effects indicated that neighboring plant performance is estimated to be reduced by 25.3% on average (Zhang et al., 2021); on the other hand, the strength of allelopathy is proposed to be much stronger if the indirect effects of plant–microbe interactions are also taken into account (Hale and Kalisz, 2012). Indeed, soil microbes are known to play critical roles in mediating allelopathic interactions between various organisms (Inderjit, 2005; Cantor et al., 2011; Xia et al., 2015; Roche et al., 2021); for example, microbes were found to be capable of regulating surrounding plants and plant communities with both negative and positive effects (Cipollini et al., 2012). In a study investigating the role of allelopathy of *Cunninghamia lanceolata*, cyclic dipeptides were discovered as active allelochemicals that were released into the soil, which not only directly poisoned the tree roots but also indirectly changed the soil microbial community composition, suggesting that the decline in productivity after continuous cultivation of *C. lanceolata* may be a negative feedback mechanism on both the allelochemical-mediated soil microbial community and root phytotoxicity (Xia et al., 2015).

Among the various hypothesis, Eppinga et al. (2006) proposed that the accumulation of local pathogens may limit the invaders abundance, but it might feed-back more negatively to the native plant community, which is conducive to the expansion of invasive plants. Soil pathogenic fungi often suppress the growth, productivity, and survival of plants, reduce the relative abundance of species in communities, mediate competitive interactions and affect succession (Mangan et al., 2010; Maron et al., 2014). A variety of invasive plants are found to be hosts of common pathogenic fungi such as *Fusarium* sp. and *Alternaria* sp. (Zhou et al., 2010; Elmer et al., 2016). *Alternaria* sp. and *Fusarium* sp. are general plant pathogens that can survive on different substrates such as water, soil and air with strong adaptability. They can cause root, stem and fruit rot or vascular wilt in a number of economically important crops, fruit trees, ornamental species, etc. (Salgado et al., 2015; Xia et al., 2020; Jindo et al., 2021). In addition, they can be replanted on the diseased host tissues to aggravate the occurrence of other diseases (Inch and Gilbert, 2003; Lawrence et al., 2008).

It has been noted that some plant species are naturally not susceptible to pathogen infection, for example *Peganum harmala* L. This plant is a medicinal herb that has a great influence on pharmacognosy and traditional medicine due to its potent therapeutic activities, such as anticancer, analgesic, hypothermic, antinociceptive, anti-inflammatory, antibacterial, antiviral and hallucinogen effects (Behidj-Benyounes et al., 2014; Bensalem et al., 2014; Li et al., 2017; Zhang et al., 2020). As a perennial species, *P. harmala* is distributed mainly in Africa, central Asia, the Middle East, South America, and the southern United States (Kartal et al., 2003; Abbott et al., 2008; Zhao et al., 2011; Zhang et al., 2020). In some areas of North America, South Australia, and South Africa, *P. harmala* is considered to be an alien invasive species that has caused not only a decline in biodiversity but also great economic losses in invaded regions (Abbott et al., 2008).

Previous physiochemical studies revealed that *P. harmala* is rich in β-carboline alkaloid derivatives such as harmine, harmaline, and harmalol; in addition, our work indicated that harmaline was the major active allelochemical responsible with potent phytotoxicity, which significantly reduced the root length of *Amaranthus retroflexus* by 47.3% at a very low concentration of 5 μg/mL (Faskhutdinov et al., 2000; Kartal et al., 2003; Shao et al., 2013). Although the production of this potent allelochemical might help explain the dominance of *P. harmala* in arid regions as well as its invasiveness in the southern United States to some extent, harmalines’ indirect allelopathic effect on soil microbes remains unclear (Abbott et al., 2008; Sodaeizadeh et al., 2009).

It is usually not easy to detect the allelopathic effects of certain allelochemicals on soil microbiota, as these effects can be rather complicated and weak. Previously, the effects of allelochemicals on soil microbial communities were mainly investigated either by pyrosequencing or cultivation approaches. Because most soil microbes are unculturable, the cultivation approach will unavoidably miss the majority of the information regarding the community structure of the soil microbiota. Although the pyrosequencing technique is developing rapidly, it still has limitations, especially with respect to the identification of microbes, as its accuracy is still unsatisfactory. Based on this, we designed a novel consecutive cultivation method (five generations of soil microbe cultivation included) to accumulate and magnify the signals triggered by harmaline so that the core information can be effectively detected. We hypothesize that harmaline released into the soil might significantly alter the community structure, diversity and composition of soil fungi that eventually favors the dominance of *P. harmala*. Based on this, the objectives of our study include: (i) investigation on the ecological effects of harmaline on the structure, diversity and composition of soil fungal communities; and (ii) determination on the growth regulatory effect of harmaline on isolated key soil fungi via *in vitro* experiments. Our study will reveal, at least in part, the contribution of harmaline to the establishment of *P. harmala*’s dominance.

## Materials and Methods

### Consecutive Cultivation Method

Soils were collected at *P. harmala* infested sites in suburban Urumqi, Xinjiang Province, China, in June 2020 (87°28’51′′, 44°33’11′′). Debris on the soil surface were first removed and then surface soils (0∼20 cm) that were at least 1 m away from the adjacent *P. harmala* patch were harvested using a sampling spade randomly from 20 different sites to avoid the presence of *P. harmala* allelochemicals. Samples were mixed thoroughly to ensure homogeneity, and impurities were removed using a 2 mm sieve. The samples were desert brown calcic soils with loose structure, poor moisture conditions, shallow soil layers, low mineral nutrient content and low organic matter content (Supplementary Table 1). Soils were then stored in a refrigerator at −80°C until required.

Before the cultivation experiment, thin layer chromatography (TLC) was applied to detect whether harmaline was present in the soil samples using chloroform: methanol = 3:1 as the developing solvent. Harmaline (purity 98%, Sigma–Aldrich Co., Shanghai) was designed to be added to the soil at a final concentration of 20 μg/g soil to investigate its effect on soil microbes. The tested concentration (20 μg/g soil) was determined according to our previous measurement of soil harmaline in *P. harmala*-infested habitats, as well as our investigation on the allelochemical concentration of other plant species, which mainly ranged from 0∼50 μg/g soil. To ensure homogeneity of harmaline in the soil matrix, 100 mg of harmaline was first dissolved in 50 mL of methanol and then added to 200 g of soil (at this point, the concentration of harmaline was 500 μg/g); the soil was then mixed thoroughly with assistance of a mortar until the methanol had completely evaporated. Next, the soil was ground into a fine powder to serve as the stock soil, which was then added to soil samples in the subsequent soil cultures to give a final concentration of 20 μg/g soil.

**FIGURE 1:**
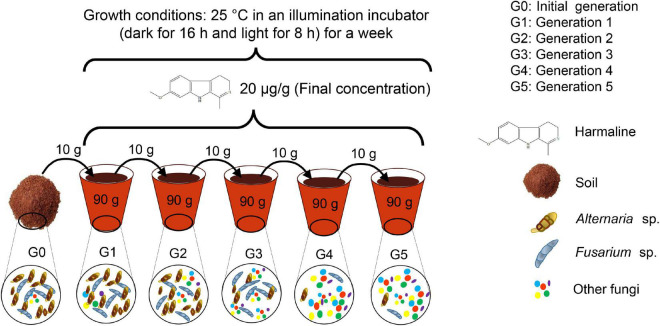
Consecutive cultivation method.

The five generations of soil cultures were prepared as follows (Figure 1). Two kilograms of collected soil was sterilized at 121°C and 0.105 MPa for 1 h, and this procedure was repeated three times on three consecutive days to obtain sterilized soil as the culture medium. The initial generation (G0) was prepared by adding 10 g of collected soil to 90 g of sterilized soil in a pot and mixing completely. Ninety grams of mixed soil was then stored in a sterilized tube at −80°C for future pyrosequencing analysis. This G0 soil represented the initial status of soil culture without adding harmaline. Then, the remaining 10 g of G0 soil was transferred to another pot, which contained 4 g of previously prepared harmaline soil (500 μg/g soil) and 86 g of sterilized soil (100 g of soil in the pot); the soils were mixed thoroughly and marked as G1, the first generation of soil culture, which included 10 g of G0 soil containing natural soil microbes and harmaline at 20 μg/g. G1 soil was kept in an Illuminated incubator under the conditions of 16/8 h. The soil microbes living in the pots were allowed to interact with harmaline at 25°C for a week; then, 90 g of G1 soil culture was removed from the pots and stored at −80°C for future use. Similarly, G2 soil was prepared by mixing 10 g of G1 soil, 4 g of harmaline soil and 86 g if sterilized soil. G3, G4, and G5 soil cultures were obtained using the same method; each generation of soil was cultured for one week as G0 soil. All treatments were performed in triplicate, and 5 weeks later, this consecutive cultivation experiment ended. Ten grams of soil samples from each generation (G0-G5) were used for pyrosequencing analysis.

### DNA Extraction and Illumina NovaSeq Sequencing

DNA was extracted from 2 g of soil using a QIAamp^®^ DNA Stool Mini Kit Handbook (QIAGEN Inc., CA, United States) according to the manufacturer’s instructions. The concentration and purity of the DNA extracts (final volume of 50 μl) were detected with a Nanodrop-NC2000 (Thermo Scientific, MA, United States); the quality of the extracted DNA was tested using 1.2% agarose gel electrophoresis. The DNA extracts were stored at –20°C for future analyses.

The ITS1 rRNA regions of isolated fungi were amplified with the pairwise common primers ITS5-1737-F (5’-GGAAGTAAAAGTCGTAACAAGG-3’) and ITS2-2043-R (5’-GCTGCGTTC TTCATCGATGC-3’) with barcodes to distinguish between the samples (White et al., 1990). The reaction mixture (25 μL) contained 5 μL of 5 × reaction buffer, 5 μL of 5 × GC buffer, 2 μL of dNTP (2.5 mM), 1 μL of forward primer (10 μM), 1 μL of reverse primer (10 μM), 2 μL of DNA template, 8.75 μL of double-distilled water and 0.25 μL of Q5 DNA polymerase. The amplification program was as follows: after an initial denaturation step at 98°C for 2 min, the targeted region was amplified by 30 cycles of 98°C for 15 s, 55°C for 30 s and 72°C for 30 s, followed by a final elongation step of 5 min at 72°C and 10°C. The PCR products were extracted from 2% agarose gels and further purified using a NovaSeq6000 SP Reagent Kit v1.5 (Illumina, Inc., San Diego, CA, United States) according to the manufacturer’s protocols. The purified amplicons were pooled in equimolar amounts, and the DNA fragments of the community were sequenced on Illumina NovaSeq platform according to the standard protocols. The raw reads were deposited in the NCBI Sequence Read Archive (SRA) database (accession number: PRJNA723748).

### Processing of the Sequencing Data

Paired-end reads were merged by the FLASH software. Low-quality sequences with a length < 300 bp and > 2 ambiguous base “N” and an average base quality score < 30 were removed. Sequence processing was conducted on an in-house pipeline. Further analyses were performed in QIIME2 (Version 2019.4) to obtain an amplicon sequence variant (ASV) table (Bolyen et al., 2019). The sequences were denoised by using DADA2, which included a strict quality control by discarding reads with ambiguous bases, singletons and chimera. Chimeras were additionally removed by using Uchime. After processing, a total of 2,127,083 effective sequences belonging to 2,992 ASVs were obtained from 18 samples. The obtained ASV table was randomly resampled with a size of 25,000 reads based on minimum sequence number per sample.

### Isolation and Identification of *Alternaria* sp. and *Fusarium* sp.

Based on the pyrosequencing results, harmaline markedly reduced the amounts of *Alternaria* sp. and *Fusarium* sp. in the soil. To further determine whether harmaline presented a negative effect on these fungi, we isolated *Alternaria* sp. and *Fusarium* sp. from *P. harmaline*-infested soils. In detail, bulk soils with *P. harmaline* collected from different sites were pooled and mixed thoroughly, then 10 g of the mixed soil was suspended in 90 mL of sterilized distilled water on a rotary shaking machine (260 rpm, 30 min) for homogenization. PDA medium was prepared by adding 40 g of potato dextrose agar (PDA) powder to 1L distilled water, which was boiled and mixed to dissolve. The medium prepared was autoclaved at 120°C for 20 min and then allowed to cool down to 50°C, followed by addition of 50 mg benzyl penicillin in order to inhibit growth of bacteria. The soil suspension was diluted from 10^–1^ to 10^–6^, followed by plating 200 μL of each suspension onto PDA medium (in 9 cm Petri dishes). The Petri dishes were incubated at 28°C for 7 days and then the colonies were isolated and purified. *Alternaria* sp. and *Fusarium* sp. were selected based on the morphological characteristics of the hyphae and spores, which were then further identified by molecular approach via DNA extraction and sequencing (ITS1, ITS4). Sequences were manually edited employing Chromas (version, 2.6.5), assembled using BioEdit (version, 7.0.9), compared for sequence homology with sequences available in NCBI (National Center for Biotechnology Information) utilizing Nucleotide BLAST (Basic Local Alignment Search Tool) and submitted to GenBank by BankIt. The results showed that ASV1 and the isolated strains AL1, AL2 and AL3 (GenBank accession numbers: MW898117, MW898118, and MW898119, respectively) were classified as *Alternaria* sp., whereas ASV2 and the isolated strains FU1, FU2, FU3 (GenBank accession numbers: MW898120, MW898121, and MW898122, respectively) were identified as *Fusarium* sp. The isolated strains were then used to examine the potential growth regulatory effects of harmaline.

### Growth Inhibitory Activity of Harmaline on Isolated *Alternaria* sp. and *Fusarium* sp.

Harmaline was dissolved in dimethyl sulfoxide (DMSO) at 10 mg/mL as the stock solution and sterilized using a 0.2 μm filter. Harmaline solutions were prepared at the following concentrations: 0, 20, 60, and 200 μg/mL using DMSO as the solvent, which was then added into sterilized PDA medium (in 9 cm Petri dishes). Mycelial plugs from the edge of the actively growing *Alternaria* sp. and *Fusarium* sp. cultures were excised and transferred to the Petri dishes, which were incubated at 28°C for 7 days. The diameters of the fungal cultures were then measured and recorded to calculate the inhibition rate of harmaline on each fungus. Four replicates were performed for each assay.

### Statistical Analysis

The sequences of the plants and algae were removed before data processing. Chao1, Pielou, Simpson and Shannon indices were first analyzed using one-way ANOVA (*p* < 0.05) to examine whether there was significant difference among the treatments, followed by multiple comparisons using the least significant difference (LSD) test (*p* < 0.05). All values were expressed as the mean ± standard error (SE). Beta diversity was estimated based on Bray–Curtis dissimilarities between samples, where the distance was computed using ASV tables. The permutational multivariate analysis of variance (PERMANOVA) with the Adonis function was used to test the differences of the fungal community composition among different treatments. We also used heatmap based on the top 20 genera normalized by Z score to compare the fungal community. The clust function was used for clustering calculations to analyze the similarity of the fungal communities in the soil cultures.

## Results

### Alpha Diversity

**FIGURE 2:**
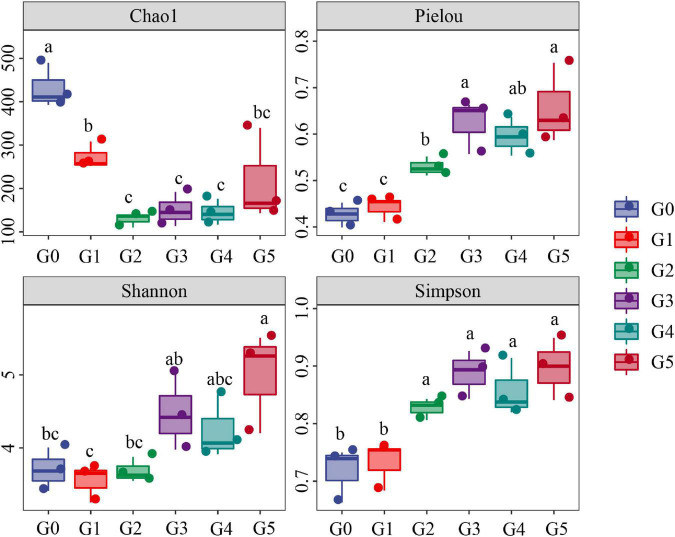
Chao1 index, Pielou index, Shannon index and Simpson index were first examined by ANOVA (*p* < 0.05) and then analyzed using Fisher’s LSD test at the *p* < 0.05 level. Means with different letters indicate significant differences at the *p* < 0.05 level according to Fisher’s LSD test.

Our results indicated a marked decline of the Chao1 index of the soil fungi in the beginning generations of culture (Figure 2). In general, Chao1 index exhibited a decreasing trend, with G1, G2, G3, G4, and G5 being reduced to 63.17, 29.93, 34.82, 33.45, and 50.13% that of G0. However, Pielou, Simpson and Shannon indices increased significantly as time went on, and were enhanced to 154.00, 134.89, and 124.97% that of G0 at the G5 stage.

### Shifts in the Fungal Community Structure

**FIGURE 3:**
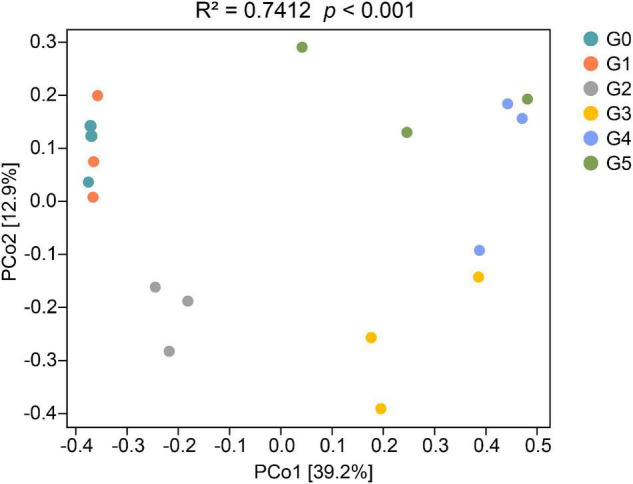
PCoA (principal coordinates PCo1 and PCo2) analysis of the fungal communities based on the Bray–Curtis distance. PCoA ordinations were analyzed by PERMANOVA.

**FIGURE 4:**
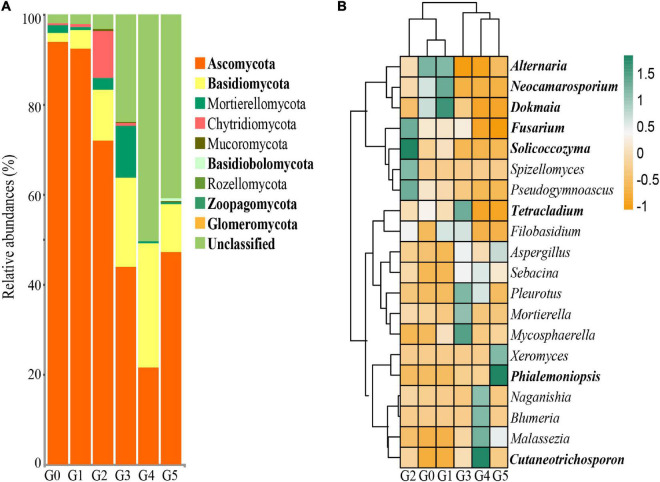
**(A)** Relative abundances of soil fungi at the phylum level. **(B)** Heatmap based on the relative abundance of the top 20 fungal genera. Bolded font indicates a significant difference at the *p* < 0.05 level by ANOVA.

The first and second axis in the PCoA ordination explained 39.2 and 12.9% of the cumulative variation of the soil fungal communities, respectively (Figure 3). According to PERMANOVA analyses, there were significant differences in the fungal communities among different generations (pseudo-*F*_5,11_ = 3.4906, *p* < 0.001). However, the heatmap containing the top 20 genera suggested that the fungal communities could be divided into two groups (Figure 4B). Cluster analysis showed that the fungal communities G0, G1, and G2 could be clustered together, whereas generations G3, G4, and G5 should be classified into another group, indicating that addition of harmaline triggered a significant effect on the soil fungal community.

At the phylum level, Ascomycota, Basidiomycota and unclassified fungi were dominant in the soils, while Mortierellomycota, Mucoromycota, Chytridiomycota and Glomeromycota were detected in the soils as less abundant fungi (Figure 4A). The relative abundances of Ascomycota, Basidiomycota, Basidiobolomycota, Zoopagomycota and unclassified fungi in G0 and G1 were significantly higher than those in G3, G4, and G5. At the genus level, significant changes in the relative abundance were also found in the soil cultures at different generations (Figure 4B). The dominant fungal genera included *Alternaria* in G0, *Alternaria, Neocamarosporium* and *Dokmaia* in G1, *Fusarium*, *Tetracladium and Solicoccozyma* in G2, *Cutaneotrichosporon* in G4, and *Phialemoniopsis* in G5, whose relative abundances were significantly higher than those of other generations.

### Suppressive Activity of Harmaline Against *Alternaria* sp. and *Fusarium* sp.

**FIGURE 5:**
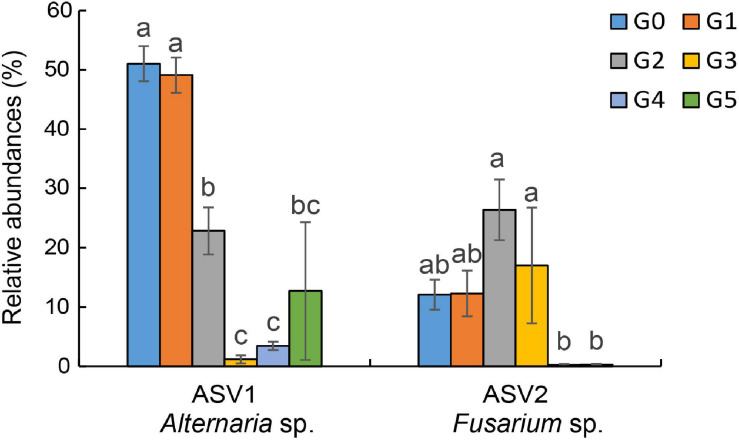
Changes in the relative abundances of ASV1 (*Alternaria* sp.) and ASV2 (*Fusarium* sp.). Different letters indicate significant differences at the *p* < 0.05 level according to Fisher’s LSD test.

**FIGURE 6:**
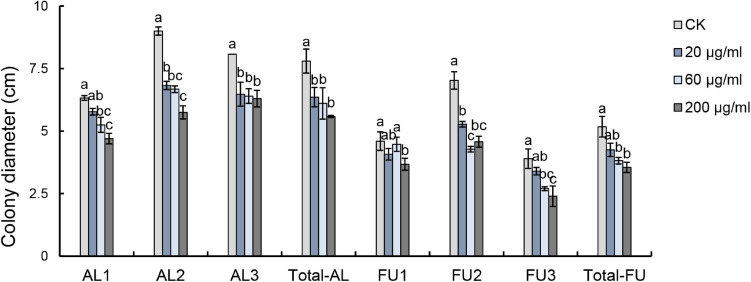
Effects of harmaline on *Alternaria* sp. and *Fusarium* sp. examined by ANOVA (*p* < 0.05) and analyzed using Fisher’s LSD test at the *p* < 0.05 level. Each value is the mean of 4 (for AL1-3, FU1-3) or 12 (for Total-AL, Total-FU) replicates ± SE. Means with different letters indicate significant differences at the *p* < 0.05 level according to Fisher’s LSD test.

Results of the pyrosequencing analysis implied that *Alternaria* sp. and *Fusarium* sp. were the most sensitive fungi affected by harmaline (Figure 5). The abundances of the dominant fungi ASV1 (*Alternaria* sp.) and ASV2 (*Fusarium* sp.) declined drastically to 84.90 and 91.90% of the control (G0), respectively. Our results showed that harmaline consistently presented a significant suppressive effect on the isolated *Alternaria* and *Fusarium* strains in a dose-dependent manner (Figure 6). Inhibitory activity was observed starting from the lowest concentration tested (20 μg/mL), which reduced the mycelia growth of AL1, AL2, AL3 by 8.70, 24.17, and 19.81%, and FU1, FU2, FU3 by 11.41, 24.91, and 12.82%, respectively. When the concentration of harmaline was increased to 200 μg/mL, growth of the *Alternaria* strains AL1, AL2, AL3 was inhibited by 25.69, 36.11, and 21.98%, and the *Fusarium* strains FU1, FU2, FU3 were affected by 20.11, 34.88, and 38.46%, respectively.

## Discussion

The novelty of our experimental design lies in its ability to magnify the signals triggered by harmaline in the soil. Inderjit and Weiner (2001) defined direct allelopathy as direct plant-plant interference mediated by allelochemicals, and indirect allelopathy as the effects of secondary compounds released by plants on abiotic and biotic soil processes that affect other plants; a number of published literature studies have indicated that direct effects appear to be much less important than the indirect effects mediated by soil ecology.

However, the physiochemical processes of allelochemicals that happen in soil, the so called black box, is rather complicated (Cortois and Deyn, 2012; Inderjit and van der Putten, 2010). In addition, plant-released allelochemicals are often influenced by various factors such as climatic conditions (e.g., temperature, precipitation, solar radiation), soil factors (e.g., organic matter content, texture, moisture, pH, ion exchanging capacity, nutrient dynamics), and plant factors of both the donor and target plants (e.g., species composition, plant diversity, growth stages) (Sun et al., 2013).

Previous works attempting to evaluate allelochemicals’ indirect effects on soil microbiota have usually been achieved by comparing the microbial community before and after the addition of certain allelochemicals (Lorenzo and Rodriguez-Echeverria, 2012; Schutz et al., 2021). However, it is quite likely that the most important signals are ignored due to the limitations of detection techniques, either via the culturing method (unfortunately, most soil microbes cannot be cultivated successfully) or pyrosequencing (which is often criticized for its unsatisfying accuracy in terms of the identification of microbes). In the current study, we transferred 1/10 of each soil as the seed soil, which was inoculated into sterilized soil that served as the culture medium to cultivate the next generation of soil microbes; in total, five generations of soil cultures were generated to accumulate the effects of harmaline on soil microbiota so that the signals could be magnified effectively. Our results indicated that in the very beginning, harmaline did not trigger a significant influence in the soil. In other words, shifts in the soil fungal community might not be noticed clearly if the consecutive cultivation approach was not adopted.

A number of published studies have pointed out that allelochemicals can pose significant impacts on soil microorganisms. For instance, phenolic acids released by *Larix gmelinii* significantly affect the total soil microbial biomass, bacteria, fungi, actinomycetes, G + bacteria, G- bacteria and AM fungi (Zhang et al., 2016). In flooded paddy soil, allelopathic rice shifted the microbial community structure owing to the release of allelochemicals such as 5,7,4-trihydroxy-3’,5’-dimethoxyflavone and benzoic acid (Gu et al., 2008). Wheat allelochemicals such as 2,4-dihydroxy-7-methoxy-1,4-benzoxazin-3-one and 6-methoxy-benzoxazolin-2-one were found to alter soil fungal populations to favor their own growth (Chen et al., 2010). Shen et al. (2020) reported that a combination of positive feedback involving allelochemicals (i.e., phenolic acids) and negative feedback involving metabolites (i.e., D-(-)-ribofuranose) contributed to the variation in soil microbial composition associated with the growth obstacles of tobacco (*Nicotiana tabacum*).

Mounting evidence has shown that allelochemicals can cause changes in specific microorganisms, which often lead to the variation of microbial community composition (Zhou et al., 2013; Arafat et al., 2020; Li et al., 2020). After the addition of phytosphingosine in the rhizosphere of *Citrullus lanatus*, the abundances of *Pseudomonas*, *Bacillus, Actinomyces*, and *Streptomyces* spp. declined significantly, leading to shifts in the community structure of the soil bacteria and fungi (Xiao et al., 2020). *Sorghum halepense* was reported to secrete p-hydroxybenzaldehyde as an effective allelochemicals to change the composition of the soil bacterial community by enhancing the abundances of *Acidobacteria*, *Chloroflexi*, *Verrucomicrobia*, and *Cyanobacteria* in the rhizosphere (Arafat et al., 2020). In another study, exogenously applied ferulic acid was found to be responsible for the significant increase of *Trichoderma* abundance in the rhizosphere soil of *Cucumis sativus* (Rahman et al., 2020). In our work, the results indicated that the soil fungal community was significantly affected by harmaline; in particular, the abundance of *Alternaria* sp. and *Fusarium* sp., eventually decreased by 84.90 and 91.90% after five generations of cultivation.

In our work, pyrosequencing analysis implied that harmaline exerted suppressive effect on the relative abundance of *Alternaria* and *Fusarium* species in the soils. A further bioassay demonstrated that indeed harmaline inhibited the mycelial growth of isolated *Alternaria* and *Fusarium* strains. Previous studies have shown that some allelochemicals are able to control plant pathogens (Saraf et al., 2014). For instance, addition of phytosphingosine decreased the abundance of *F. oxysporum* (Li et al., 2020). Glucosinolate-derived isothiocyanates produced by *Brassica* species was found to suppress the growth of a number of soil-borne pathogenic fungi such as *Gaeumannomyces graminis*, *Rhizoctonia solani*, and *Fusarium* sp. (Sarwar et al., 1998; Taylor et al., 2014). Notably, plant-derived alkaloids have been suggested to possess antimicrobial activity. The alkaloid securinine produced by *Phyllanthus amarus* was reported to inhibit pathogenic fungi such as *A. brassicae, A. brassicicola, A. solani*, and *F. udum*, etc. (Singh et al., 2008). Potato steroidal alkaloids and their nanoparticles were also found to be able to control the mycelial growth of phytopathogenic fungi including *A. alternate*, *R. solani*, *Botrytis cinerea*, and *F. oxysporum*. In another study, the alkaloid 1-corydalmine suppressed spore germination of *A. brassicae* and *F. udum* (Basha et al., 2007; Almadiy and Nenaah, 2018). Alkaloids have been found to be capable of destroying cell walls and the membrane structures of fungal cells, thus achieving fungal inhibition (Moraes et al., 2015; Wei et al., 2020).

*Peganum harmala* has been reported to be able to produce a relatively large amount of harmaline that can be detected in its seeds, stems, leaves, roots and rhizosphere soil (Faskhutdinov et al., 2000; Bensalem et al., 2014; Li et al., 2018). Our results suggest that harmaline can effectively inhibit growth of the general pathogens *Alternaria* sp. and *Fusarium* sp., which might explain the phenomenon that diseased *P. harmala* plants are rarely found in natural habitats. We suppose that the presence of harmaline in *P. harmala* can be depicted as a carry-on pesticide to defend against general pathogens. It can outcompete other native species without the ability to produce bioactive fungicidal substances. This mechanism might contribute, at least in part, to the establishment of *P. harmala*’s dominance as a successful native species.

## Conclusion

Our consecutive cultivation procedure allowed the allelochemical harmaline to function for longer duration at effective concentration so that signals of the shifts in the soil microbiota can be accumulated and magnified. Our results indicated that harmaline altered the community composition of soil fungi; in particular, harmaline reduced the relative abundances of the general pathogenic fungi *Alternaria* sp. and *Fusarium* sp. so as to improve the soil condition. Further *in vitro* bioassays confirmed that harmaline indeed suppressed the mycelial growth of six isolated *Alternaria* and *Fusarium* strains, implying that harmaline has the potential to be further utilized as a soil amendment agent to improve soil quality. We thus suspect that *P. harmala* might produce harmaline as an effective carry-on pesticide to defend against general pathogens and outcompete other neighboring species to facilitate the establishment of *P. harmala*’s dominance.

## Data Availability Statement

The datasets presented in this study can be found in online repositories. The names of the repository/repositories and accession number(s) can be found in the article/Supplementary Material.

## Author Contributions

KS: conceptualization, methodology, software, validation, formal analysis, investigation, resources, data curation, and writing original draft preparation. HS: writing – review and editing, visualization, supervision, project administration, and funding acquisition. Both authors contributed to the article and approved the submitted version.

## Conflict of Interest

The authors declare that the research was conducted in the absence of any commercial or financial relationships that could be construed as a potential conflict of interest.

## Publisher’s Note

All claims expressed in this article are solely those of the authors and do not necessarily represent those of their affiliated organizations, or those of the publisher, the editors and the reviewers. Any product that may be evaluated in this article, or claim that may be made by its manufacturer, is not guaranteed or endorsed by the publisher.
