# Supplementary material for: Changes in the Soil Fungal Community Mediated by a Peganum harmala Allelochemical
Source: Front Microbiol. 2022 Jun 16;13:911836. doi: 10.3389/fmicb.2022.911836 (PMC9243656; doi:10.3389/fmicb.2022.911836)
Supplement: Supplementary file 1 [file Table_1.DOCX]

Supplementary Material

# Supplementary Tables

**Supplementary Table1.** Physicochemical properties of collected soils

| Soil nutrients | SOC (g/kg) | SOM (g/kg) | TN (g/kg) | TP (g/kg) | TK (g/kg) | AN (mg/kg) | AP (mg/kg) | AK (mg/kg) | pH |
| --- | --- | --- | --- | --- | --- | --- | --- | --- | --- |
| Mean contents | 3.21 | 5.84 | 0.41 | 0.49 | 14.38 | 27.21 | 5.08 | 77.35 | 8.58 |

Note: SOM: soil organic matter (SOM); SOC: soil organic carbon; TN: total nitrogen; TP: total phosphorous; TK: total potassium; AN: available nitrogen; AP: available phosphorous; AK: available potassium. Each value is the average of 3 replicates (n = 3).
